# Annexin A3, a Calcium-Dependent Phospholipid-Binding Protein: Implication in Cancer

**DOI:** 10.3389/fmolb.2021.716415

**Published:** 2021-07-20

**Authors:** Liu Yang, Pingan Lu, Xiaohui Yang, Kaiguo Li, Song Qu

**Affiliations:** ^1^ Key Laboratory of High-Incidence Tumor Prevention and Treatment (Guangxi Medical University), Ministry of Education, Department of Radiation Oncology, Guangxi Medical University Cancer Hospital, Nanning, China; ^2^ Faculty of Medicine, Amsterdam Medical Centre, University of Amsterdam, Amsterdam, Netherlands

**Keywords:** annexin A3, biomarkers, drug resistance, neoplasm, signal transduction, tumorigenesis

## Abstract

Annexin A3 (ANXA3), also known as lipocortin III and placental anticoagulant protein III, has been reported to be dysregulated in tumor tissues and cancer cell lines, and harbors pronounced diagnostic and prognostic value for certain malignancies, such as breast, prostate, colorectal, lung and liver cancer. Aberrant expression of ANXA3 promotes tumor cell proliferation, invasion, metastasis, angiogenesis, and therapy resistance to multiple chemotherapeutic drugs including platinum-based agents, fluoropyrimidines, cyclophosphamide, doxorubicin, and docetaxel. Genetic alterations on the ANXA3 gene have also been reported to be associated with the propensity to form certain inherited, familial tumors. These diverse functions of ANXA3 in tumors collectively indicate that ANXA3 may serve as an attractive target for novel anticancer therapies and a powerful diagnostic and prognostic biomarker for early tumor detection and population risk screening. In this review, we dissect the role of ANXA3 in cancer in detail.

## Introduction

Annexin A3 (ANXA3), a water-soluble protein consisting of 323 amino acid residues, is encoded by the ANXA3 gene located on human chromosome 4q13–q22 (Mussunoor and Murray, 2008). Also known as lipocortin III and placental anticoagulant protein II, ANXA3 subordinate to the annexin family, which is a well-characterized multigene family of structurally homologous, but functionally different calcium-dependent membrane phospholipid-binding proteins ubiquitously distributed in a wide array of cell types (Moss and Morgan, 2004).

The annexin family can be divided into five classes (A-E) based on their biological origins; among them, 12 subtypes of class A annexins, annexins A1 to A11 and A13, are derived from human and vertebrate orthologues, while class B till E are originated from non-vertebrate metazoans, fungi and molds, plants and protists respectively (Gerke and Moss, 2002). ANXA3 has been demonstrated to be virtually solely expressed in differentiated myeloid cell lines and accounts for about 1% of the cytosolic protein of human neutrophils (Sopkova et al., 2002). To date, two isoforms of ANXA3 with the molecular mass of 33 and 36 kDa have been documented. The 36 kDa ANXA3 isoform is mainly expressed in monocytes, while the 33 kDa ANXA3 isoform is more abundantly observed in neutrophils (Le Cabec and Maridonneau-Parini, 1994).

**FIGURE 1:**
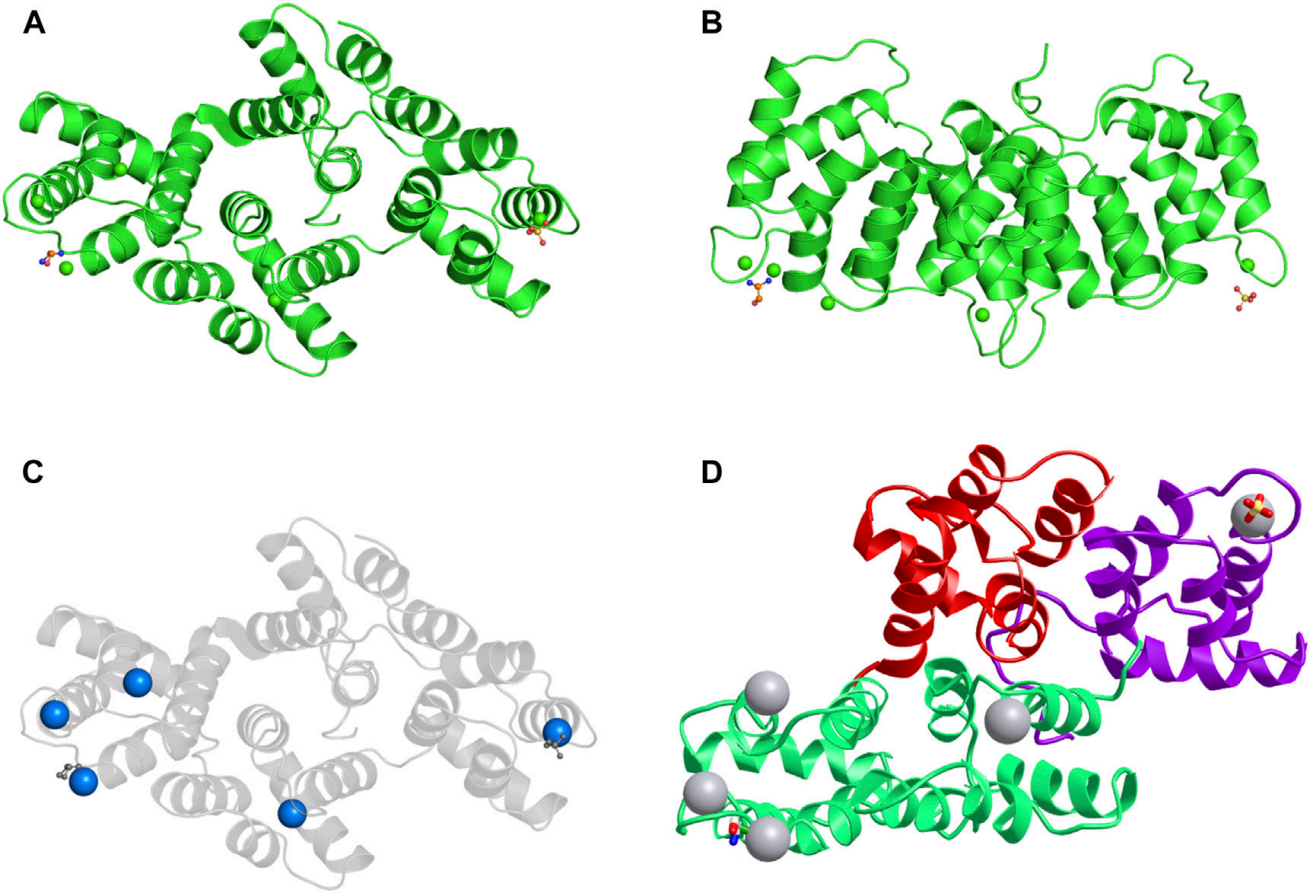
The structure of the ANXA3 protein. **(A,B)** 3D structure of ANXA3 colored by chain and viewed from the front **(A)** and side **(B)**. **(C)** 3D structure viewed from front with calcium-binding sites indicated by calcium ions colored as blue spheres. **(D)** 3D structure colored by domain. 5 *a*-helices are clearly visible in each structural domain. The remaining two chemical structures are one sulfate ion and one ethanolamine. Data derived from Protein Data Bank and iCn3D web-based 3D structure viewer.

Four conserved annexin repeats structural domains (I-IV) constitute the C-terminal protein core of ANXA3 (Sopkova et al., 2002) (Figure 1). Each structural domain encompasses five *a*-helices (A-E) consisting of 70 amino acid residues (Favier-Perron et al., 1996; Gerke and Moss, 2002). In addition, one principal calcium-binding site is present on the convex face formed by *a*-helices A and B in each structural domain (Raynal and Pollard, 1994; Mussunoor and Murray, 2008). The highly variable N-terminus of 20 amino acids in length contains two tryptophan residues, which are W5 in the N-terminal segments and W190 at the end of the IIIA-IIIB loop (Sopkova et al., 2002). These two tryptophan residues are essential for the protein stability as well as the interaction of ANXA3 with intracellular calcium ions and negatively charged phospholipids, thereby regulating a diverse range of biological functions of ANXA3 (Hofmann et al., 2000; Sopkova et al., 2002). Due to its phospholipid-binding capacity and calmodulin-dependent nature, ANXA3 primarily participates in membrane-associated activities, such as intracellular and extracellular signal transduction, vesicular transport, membrane fusion and endocytosis, formation and transport of ion channels, and interactions of cytoskeleton proteins (Swairjo and Seaton, 1994; Perron et al., 1997; Mussunoor and Murray, 2008). The involvement of ANXA3 in cellular signal transduction facilitates its multifaceted regulatory roles in various physiological activities, including cell division, differentiation, motility and apoptosis as well as anti-inflammation, anticoagulation and angiogenesis (Moss and Morgan, 2004; Mussunoor and Murray, 2008). Meanwhile, dysregulation of ANXA3 has been reported to play a pivotal role in cancer development and progression (Mussunoor and Murray, 2008) (Table 1 and Figure 4). However, data published so far about its expression in different malignancies are inconsistent. To the best of our knowledge, ANXA3 has been reported to be overexpressed in a majority cancer types including breast (Pendharkar et al., 2016; Zhou et al., 2017a; Zhou et al., 2017b; Guo et al., 2017; Aravind Kumar et al., 2018; Du et al., 2018; Kim et al., 2018; Li et al., 2018; Zhou et al., 2018), colorectal (Madoz-Gurpide et al., 2006; Marshall et al., 2010; Yip et al., 2010; Yang L. et al., 2018; Yang Q. et al., 2018; Xu et al., 2019), bladder (Tsai et al., 2018), ovarian (Jiang et al., 2019), gastric (Takahashi et al., 2015; Wang and Li, 2016) and pancreatic cancer (Baine et al., 2011a; Baine et al., 2011b; Wan et al., 2020) as well as hepatocellular (Pan et al., 2015a; Pan et al., 2015b; Tong et al., 2018) and nasopharyngeal carcinoma (Ruan et al., 2010), while downregulated in renal (Bianchi et al., 2010), prostate (Wozny et al., 2007; Köllermann et al., 2008; Peraldo-Neia et al., 2011) and papillary thyroid cancer (Jung et al., 2010). Furthermore, the expression of ANXA3 in lung cancer remains controversial, with the evidence of both upregulated (Liu et al., 2009; Győrffy et al., 2013; Wang et al., 2019; Jin et al., 2020; Liu et al., 2021) and downregulated (Rho et al., 2009; Wu et al., 2018; Lohinai et al., 2019) expression patterns documented in the literature. In an immunohistochemistry-based study of organotypic *ex vivo* human HCC clinical samples and HCC patient-derived xenografts, Tong et al. found that overexpression of ANXA3 was associated with enhanced resistance to sorafenib and led to poor survival of HCC patients receiving sorafenib treatment. Their data further indicates that targeting ANXA3 could effectively inhibit tumor growth and sensitize the response of tumor cells to sorafenib treatment (Tong et al., 2018). In addition, ANXA3 mRNAs and proteins were overexpressed in gastric cancer tissues and various gastric cancer cell lines, as detected by RT-PCR and Western blot analyses (Wang and Li, 2016). This aberrant expression was further correlated with the depth of tumor infiltration and TNM stage in both univariate and multivariate analyses of a cohort of 183 gastric cancer patients, which indicates the potential of ANXA3 as an independent prognosticator for the survival of gastric cancer patients (Wang and Li, 2016). Likewise, markedly elevated ANXA3 expression was detected in bladder cancer by multiplexed liquid chromatography multiple-reaction-monitoring mass spectrometry assay (LC-MRM-MS). Investigators from this study further suggested that ANXA3 might serve as a reliable non-invasive diagnostic biomarker for bladder cancer (Tsai et al., 2018). Similarly, ANXA3 was overexpressed in colorectal cancer (CRC) tissues compared to adjacent normal tissues, as shown from immunohistochemistry and western blot results (Yang Q. et al., 2018). Moreover, a Max Vision immunohistochemistry-based retrospective analysis of a cohort of 309 breast cancer patients demonstrated that ANXA3 expression in triple negative breast cancer (TNBC) patients was significantly higher than other breast cancer subtypes (Zhou et al., 2017b). Given that overexpression of ANXA3 has a vital impact on tumor progression, we could expect that downregulation of ANXA3 can also exert certain regulatory effects on tumorigenesis. Interestingly, ANXA3 expression level was diminished in prostate tumor tissues and was correlated with increasing pathological stages and Gleason scores (Köllermann et al., 2008). Immunohistochemistry and tissue microarray data further confirmed that ANXA3 could be used as an independent prognostic factor to predict the survival of prostate cancer patients and to support population risk stratification (Köllermann et al., 2008). Downregulation of ANXA3 was also reported in papillary thyroid cancer (PTC), and PTC patients with decreased ANXA3 expression exhibited substantially elevated lymph node metastasis scores and tumor growth (Jung et al., 2010).

**TABLE 1:** The role of ANXA3 in tumor proliferation, invasion and metastasis.

Types	Expression	Models	Evidence	References
Hepatocellular carcinoma (HCC)	↑	6 cell lines and HCC tumor tissues	ANXA3 is overexpressed in sorafenib-resistant HCC cells, which inhibits PKCδ/p38-associated apoptosis and stimulates p38-mediated autophagy for cell survival	Tong et al. (2018)
	↑	7 cell lines; Tissues from 83 patients	Endogenous and secretory ANXA3 promotes tumor growth and stemness acquisition through dysregulating JNK pathway	Tong et al. (2015)
	↑	4 cell lines; 107 patients with HCC	The expression of HIF-1a, CD133, Notch1, Notch2 is significantly increased in ANXA3-overexpressing cells. Overexpression of ANXA3 enhances the proportion and tumorigenicity of CD133 + cells	Pan et al. (2015a)
	↑	Sk-hep-1 and SMMC-7721 cell line	Small interfering RNA silencing ANXA3 inhibits tumorigenesis and metastasis	Pan et al. (2015b)
Breast cancer	↑	MDA-MB 231 cell line and tissue from 30 primary breast cancer patients	ANXA3 knockdown inhibits proliferation, invasion, migration, and colony formation of tumor cells	Kim et al. (2018)
	↑	18 female nude mice inoculated with MDA-MB-231 cells	Breast cancer cells transfected with ANXA3 silencing shRNA exhibit significantly lower tumor weight, volume and tumorigenic activity	Li et al. (2018)
	↑	Samples from 158 patients; Tissue specimens	Enhanced cell proliferation indexes are positively correlated with the ANXA3 mRNA and protein expression level	Zhou et al. (2018)
	↑	16 pairs of breast cancer tissues and adjacent normal tissues; 3 cell lines	Silencing ANXA3 suppresses the NFκB pathway via upregulating IκBα, leading to mesenchymal-epithelial transition (MET) with attenuated invasion and metastasis, but promotes tumor cell proliferation	Du et al. (2018)
	↑	2 cell lines	Migration and invasion ability is lower in ANXA3 silenced cells	Zhou et al. (2017a)
	↑	309 breast cancer patients and their tissue specimens	ANXA3 is correlated with increased number of lymphatic metastases and advanced histological grading	Zhou et al. (2017b)
Lung carcinoma	↑	3 cell lines	Activation of the ANXA3/JNK pathway inhibits cisplatin-induced apoptosis	Wang et al. (2019)
	↑	Excised tissues from 21 lung AdC patients	ANXA3 expression is positively correlated with lymph node metastasis and the clinicopathological stages of lung adenocarcinoma (AdC)	Liu et al. (2009)
	↑	5 cell lines; 102 LC tissues and 102 paracancerous tissues	Knockdown of ANXA3 expression by miR-1253 inhibited proliferation, invasion and increased apoptosis rate	Liu et al. (2021)
Colorectal cancer (CRC)	↑	Tumor tissues from 107 CRC patients; 5 cell lines	miR-340-5p directly targets ANXA3, resulting in enhanced CRC cell proliferation, migration, and invasion	Yang et al. (2018a)
	↑	2 cell lines	ANXA3 depletion inhibits proliferation and facilitates apoptosis in oxaliplatin-resistant cells	Xu et al. (2019)
Pancreatic cancer	↑	Sample from 115 PC patients; 4 cell lines	ANXA3 knockdown using microRNA-382 inhibits PI3K/AKT signaling pathway, give rise to suppression of pancreatic cancer proliferation, invasion and metastasis	Wan et al. (2020)
Gastric cancer	↑	Tissues from 183 GC patients; 5 cell lines	ANXA3 depletion suppresses cell proliferation, invasion and metastasis	Wang and Li, (2016)
Ovarian cancer	↑	OC tissues; Normal ovarian cell line and OC cell lines	Overexpression ANXA3 leads to augmented proliferative and migratory behavior	Jiang et al. (2019)
Papillary thyroid carcinoma	↓	25 patients-derived tissue specimens	Reduced ANXA3 immunohistochemical staining is correlated with tumors with higher lymph node metastasis scores and larger sizes	Jung et al. (2010)

Collectively, aberrant expression of ANXA3 plays a crucial role in malignant tumor development. It stimulates tumor cell proliferation, facilitates invasion, migration and metastasis, induces angiogenesis, desensitizes patient response to antitumor treatments and predisposes the emergence of certain inherited familial tumors (Gerke and Moss, 2002; Moss and Morgan, 2004; Mussunoor and Murray, 2008; Tong et al., 2018; Sarquis et al., 2020). Therefore, it is important to shedding light on the functions of ANXA3 in tumor biology in order to improve the early detection of preneoplastic tumors, to overcome anticancer therapy resistance and to develop novel, targeted approaches to treat solid tumors. This review focusses on the roles of ANXA3 in cancer.

## The Role of ANXA3 in Tumorigenesis

### Sustaining Proliferative Signaling

**FIGURE 2:**
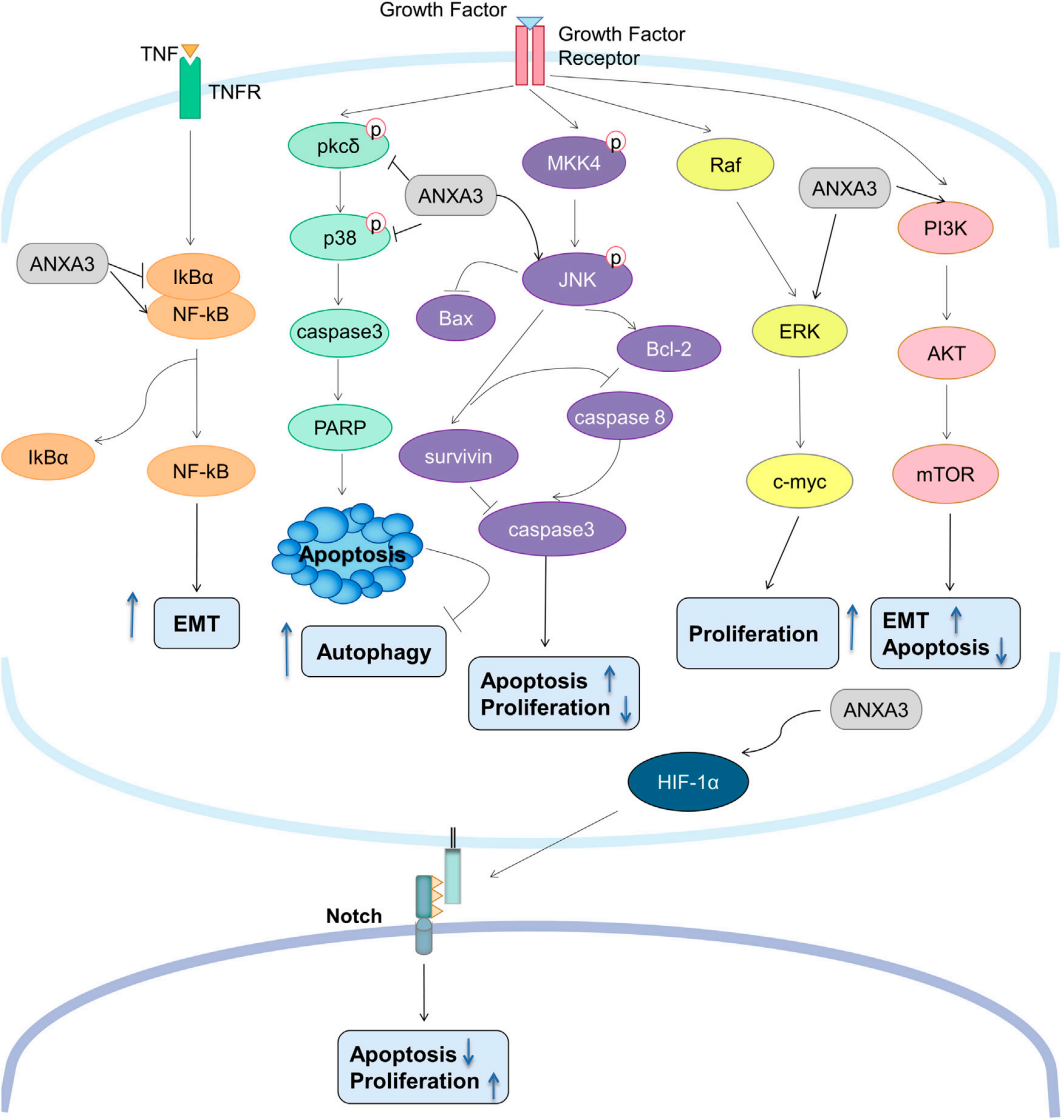
Overview of the signal transduction network of ANXA3 in tumors. ANXA3 promotes tumorigenesis *via*: (1) activating NFkB pathway leading to increased EMT; (2) inhibiting PKCδ/p38 pathway leading to decreased apoptosis and increased autophagy; (3) activating JNK/survivin and Raf/ERK/c-myc pathways leading to increased proliferation; (4) activating PI3K/Akt/mTOR pathway leading to increased EMT and decreased apoptosis; and (5) activating HIF-1α/Notch pathway leading to increased proliferation and decreased apoptosis. p38, p38 mitogen-activated protein kinases; PARP, Poly (ADP-ribose) polymerase; bax, Bcl-2-associated X protein; bcl-2, B-cell lymphoma 2; IκBα, nuclear factor of kappa light polypeptide gene enhancer in B-cells inhibitor, alpha; NF-κB, nuclear factor kappa-light-chain-enhancer of activated B cells; HIF-1α, Hypoxia-inducible factor 1-alpha; EMT, Epithelial-Mesenchymal Transition; TNF, Tumor necrosis factor.

Sustaining proliferative signaling is a common strategy used by cancer cells to facilitate their progression and aggressiveness. Recent years, the pro-proliferative role of ANXA3 has been substantiated to be heavily implicated in various types of malignancies, and a diversity of evidence has been provided about the underlying mechanisms mediating this process (Figure 2). In hepatocellular carcinoma, ANXA3 has been shown to activate the Notch and MAPK/ERK/JNK signaling pathway, resulting in enhanced cell proliferation and promotion of stem-cell like characteristics (Pan et al., 2015a; Tong et al., 2015). Support on this finding was delivered by *in vitro* data of colorectal cancer, in which the phosphorylation of ERK and JNK was found to be significantly reduced once a depletion of ANXA3 was established using small interfering RNA (siRNA) (Xu et al., 2019). This finding was further corroborated by another study on chemoresistant non-small cell lung cancer (NSCLC) cells, which showed that high level of ANXA3 secreted by cancer associated fibroblasts (CAFs) in the tumor microenvironment activated the JNK/survivin signaling, thereby helping cancer cells escaping the cisplatin-induced apoptosis (Wang et al., 2019). Another putative mechanism contributing to the pro-proliferative effect of ANXA3 was provided by the work of Wan et al., in which PI3K/Akt signaling pathway was found to be substantially inhibited in pancreatic cancer patients with overexpression of miR-382, a miRNA that suppresses the expression of the ANXA3 gene (Wan et al., 2020). Investigators in this study further observed a decline of clone formation ability and proliferative behavior in pancreatic cancer cells overexpressing miR-382. Interestingly, a research on breast cancer has described an opposite correlation; the depletion of ANXA3 using short hairpin RNA plasmids has been shown to promote cell proliferation in both cell-line models and mouse xenograft models (Du et al., 2018). However, a series of studies on breast cancer challenged this finding. Collectively, these studies demonstrated that ANXA3 is highly expressed in luminal A, B and triple negative breast cancer subtypes, and that ANXA3 inhibition could significantly impair tumor growth *in vivo*, concomitant with a lower proliferation index and a higher apoptosis rate and G0/1 cell count *in vitro* (Zhou et al., 2017a; Li et al., 2018; Zhou et al., 2018).

Aside from promoting the pro-proliferative pathways, aberrant expression of ANXA3 has also been shown to downregulate multiple pro-apoptotic proteins and cyclin-dependent kinases (CDKs), which facilitates the evasion of apoptosis and cell cycle arrest. For example, *in vitro* investigations on breast cancer cell lines HCC-1954 and MDA-MB-231 demonstrated that ANXA3 silencing using siRNA significantly reduced the expression of CDK4 and enhanced the expression of E2F1 and p27^Kip1^ (Kim et al., 2018). Furthermore, high levels of ANXA3 in hepatocellular carcinoma has been demonstrated to attenuate the PKCδ/p38 associated apoptosis (Tong et al., 2018). Of note, p38 not only plays a role in the regulation of apoptosis, but also acts a key regulator in autophagy, which is another defining feature of tumor cells that supplies metabolic fuel sources for unlimited proliferation (Webber, 2010; Webber and Tooze, 2010). Unsurprisingly, investigators from this study further detected a substantially increased level of autophagic marker LC3B in both HCC cells *in vitro* and mouse xenografts *in vivo*, thereby confirming the positive correlation between ANXA3 expression and autophagic activity (Tong et al., 2018). The anti-apoptotic ability of ANXA3 was further substantiated by the work of Wang et al., wherein pro-apoptotic proteins caspase 3 and caspase 8 was significantly downregulated in NSCLC cells overexpressing ANXA3 (Wang et al., 2019). Conversely, miRNA-induced silencing of ANXA3 markedly upregulated caspase 3 expression as well as the expression of pro-apoptotic protein Bax, while suppressing the expression anti-apoptotic protein Bcl-2 (Liu et al., 2021). Consistent data were also published by Xu et al., who observed an ANXA3 knockdown-induced upregulation of c-caspase 3 and c-PARP in colorectal cancer cells (Xu et al., 2019).

### Promoting Invasion and Metastasis

Invasion and metastasis are the major cause of poor clinical outcomes of malignant diseases. *In vitro* investigations have revealed that ANXA3 overexpression significantly stimulated the invasion and migration of breast cancer cells (Ibrahim et al., 2012; Guo et al., 2017; Kim et al., 2018). Clinically, ANXA3 overexpression has been demonstrated to be correlated with the occurrence of lymph node metastasis and the clinicopathological stages of breast cancer (Zhou et al., 2017b) and lung adenocarcinoma (Liu et al., 2009). The correlation was further reversely validated by the work of Zhou et al., in which knockdown of ANXA3 by shRNA impaired the invasion and migration abilities of luminal A and triple negative breast cancer cells (Zhou et al., 2017a). Similar approaches employing miRNA and siRNA to probe the effect of ANXA3 depletion have also been applied in *in vitro* cell-line models of colorectal carcinoma (Yang L. et al., 2018; Xu et al., 2019) and ovarian carcinoma (Jiang et al., 2019), which presented consistent results. Intriguingly, an inverse relationship between ANXA3 expression and the execution of invasion-metastasis cascade in malignant tumors has also been documented. In surgical tumor specimens from 25 thyroid papillary cancer patients receiving thyroidectomy, downregulation of ANXA3 was detected in tumor tissues compared to the adjacent non-tumor tissues (Jung et al., 2010). Further immunohistochemistry results showed that reduced ANXA3 staining was correlated with thyroid papillary tumors with higher lymph node metastasis scores and larger sizes (Jung et al., 2010). Nonetheless, no data are currently available about what factors govern the downregulated expression of ANXA3 and its potentially anti-oncogenic activities in thyroid neoplasms.

A number of attempts has been made to decipher the underlying molecular changes mediating the ANXA3-induced invasion and migration. In ANXA3 overexpressing gastric cancer cells and patient-derived tumor specimens, an enhanced degree of epithelial-mesenchymal transition (EMT) was observed, which was evidenced by western blot results indicating increased expressions of mesenchymal markers vimentin and *ß*-catenin, a decreased expression of epithelial marker E-cadherin, and increased expressions of EMT-related transcription factors fibronectin, Slug and Snail (Wang and Li, 2016). Contrariwise, silencing of ANXA3 inhibited the expression of N-cadherin and vimentin in pancreatic cancer, while elevating the expression of E-cadherin (Wan et al., 2020). Additionally, ANXA3-knockdown pancreatic cancer cells exhibited decreased expressions of VEGF-C and VEGF-D (Wan et al., 2020), both of which are proteins previously shown to be positively associated with the number of lymph node metastases (Schulz et al., 2011). Similar findings were reported by research involving triple negative breast cancer cell lines MDA-MB-231 and MDA-MB-486, which found mesenchymal-epithelial transition (MET) in cancer cells receiving ANXA3-targetting shRNA plasmids, evidenced by decreased mesenchymal markers (vimentin and N-cadherin) and increased epithelial markers (E-cadherin and *γ*-cadherin) (Du et al., 2018). This reversed transition pattern could be considered as an indicator of diminished invasion and migration abilities. Interestingly, this study further indicated that IκBα knockdown could partially reverse the ANXA knockdown-induced MET state concomitant with an increased phospho-NF-κB p65 expression. These findings suggest that NF-κB signaling might be crucially involved in the ANXA3-medicated tumor cell invasion and migration (Du et al., 2018).

### Inducing Angiogenesis

Angiogenesis is the essential mechanism that allows continuous nutrients supply and assists tumor cells to combat hypoxia in the tumor microenvironment. Over the last decade, a series of studies has identified novel clues shedding light on the functional link between ANXA3 and tumor angiogenesis. At histological level, less blood vessels were observed in H&E-stained tissue slides of triple negative breast cancer xenografts from mice pretreated with ANXA3-silencing shRNA plasmids (Li et al., 2018). At molecular level, ANXA3 silencing in pancreatic cancer cells resulted in a decrement of the expression of vascular endothelial growth factor receptor 3 (VEGFR3), which is a protein suggested to play a key role in lymphatic vascularization in pancreatic cancer (Wan et al., 2020). In addition, investigators from this study observed an ANXA3-induced upregulation of PI3K/Akt signaling pathway, which is a canonical pathway also capable of promoting neovascularization apart from its pro-proliferative effects (Sun et al., 2016; Wan et al., 2020). The hypoxia-inducible factor-1α (HIF-1α)/VEGF pathway is another well-established response of cancer cell to initiate angiogenesis and thereby survive hypoxia (Xu et al., 2018; Zhang et al., 2018; Zhang P.-C. et al., 2020). Employing liver cancer stem-like cells, Pan et al. revealed a positive correlation between the expressions of ANXA3 and HIF1α, which further confirmed the pro-angiogenic role of ANXA3 (Pan et al., 2015a). Similar findings were also presented by a research on bone-cancer induced pain (BCP), showing that downregulation of ANXA3 using shRNA substantially inhibited the expression of HIF1α and VEGF in the ipsilateral spinal cord and microglial cells of mice undertaken 21 days of bone cancer induction (Zhang Z. et al., 2020). Besides cancer-related investigations, fundamental studies using human umbilical vein endothelial cell (HUVEC) model provided extra confirming data (Park et al., 2005; Mohr et al., 2017). Employing the hypoxia responsive element (HRE)-luciferase reporter assay, investigators demonstrated that ANXA3 upregulated the HIF1α transactivation activity and VEGF production, give rise to enhanced tube formation and migration of HUVECs(Park et al., 2005). These findings suggest that ANXA3 might act as a key driver in angiogenic processes.

Paradoxically, a negative relationship between the expressions of ANXA3 and HIF1α has been reported in renal cell carcinoma (RCC) (Bianchi et al., 2010). Whereas RCC cultures not expressing HIF1α (HIF1α-negative) exhibited a similar level of ANXA3 expression as the matched noncancerous cortex, a markedly decreased ANXA3 expression was detected in HIF1α-positive RCC cultures compared to the matched counterparts (Bianchi et al., 2010). Moreover, the abundance of 36-kDa ANXA3 was significantly reduced in HIF1α-positive RCC cultures, while the 33-kDa ANXA3 showed a pronounced increment (Bianchi et al., 2010). Most importantly, the total expression of ANXA3 protein was significantly lower in RCC cultures *in vitro* as well as in surgical excised RCC tissues compared to their paired counterparts (Bianchi et al., 2010). It was speculated that the downregulation of the total ANXA3 protein in RCC was associated with the decrement of its 36-kDa isoform, as the N-terminus of ANXA3 capable of promoting self-expression is present in the 36-kDa isoform but absent in the 33-kDa isoform (Hofmann et al., 2000; Gerke et al., 2005; Bianchi et al., 2010). However, the mechanism underlying the diminution of 36-kDa ANXA3 in RCC remains unclear, and the differences between the two isoforms regarding their expressions and functions in other cancer models are still far from fully understood. 

### Genetic Predisposition

Genetic predisposition usually serves as a prerequisite in cancer initiation and harbors pronounced clinical relevance especially in aggressive malignancies where an urgent need exists for novel risk screening methods with good predictive performance and clinical utility. Cancer-associated mutations can not only drive accelerated cancer progression but can also exhibit inherited patterns, contributing to familial hereditary tumors (AlHarthi et al., 2020). So far, very limited evidence has been published reporting the association between genetic alterations of the ANXA3 gene with cancer susceptibility. In a case-control study of 29 TNBC patients searching for risk-associated SNPs through microarray-based whole genome SNP sequencing, the non-synonymous SNP exm4087722 was detected in the ANXA3 gene (NM_005139) with a minor allele frequency (MAF) smaller than 0.05 (Aravind Kumar et al., 2018). Whole genome sequencing was also applied in another research on 3 Brazilian families with hereditary papillary thyroid cancer (PTC), which found point mutation p.D283N on the ANXA3 gene (MIM No.106490) (Sarquis et al., 2020). Yet, it is important to note that above observations are both based on small sample sizes and that more conclusive data from larger cohorts are still awaited about the role of genetic alterations of the ANXA3 gene in cancer susceptibility.

**FIGURE 3:**
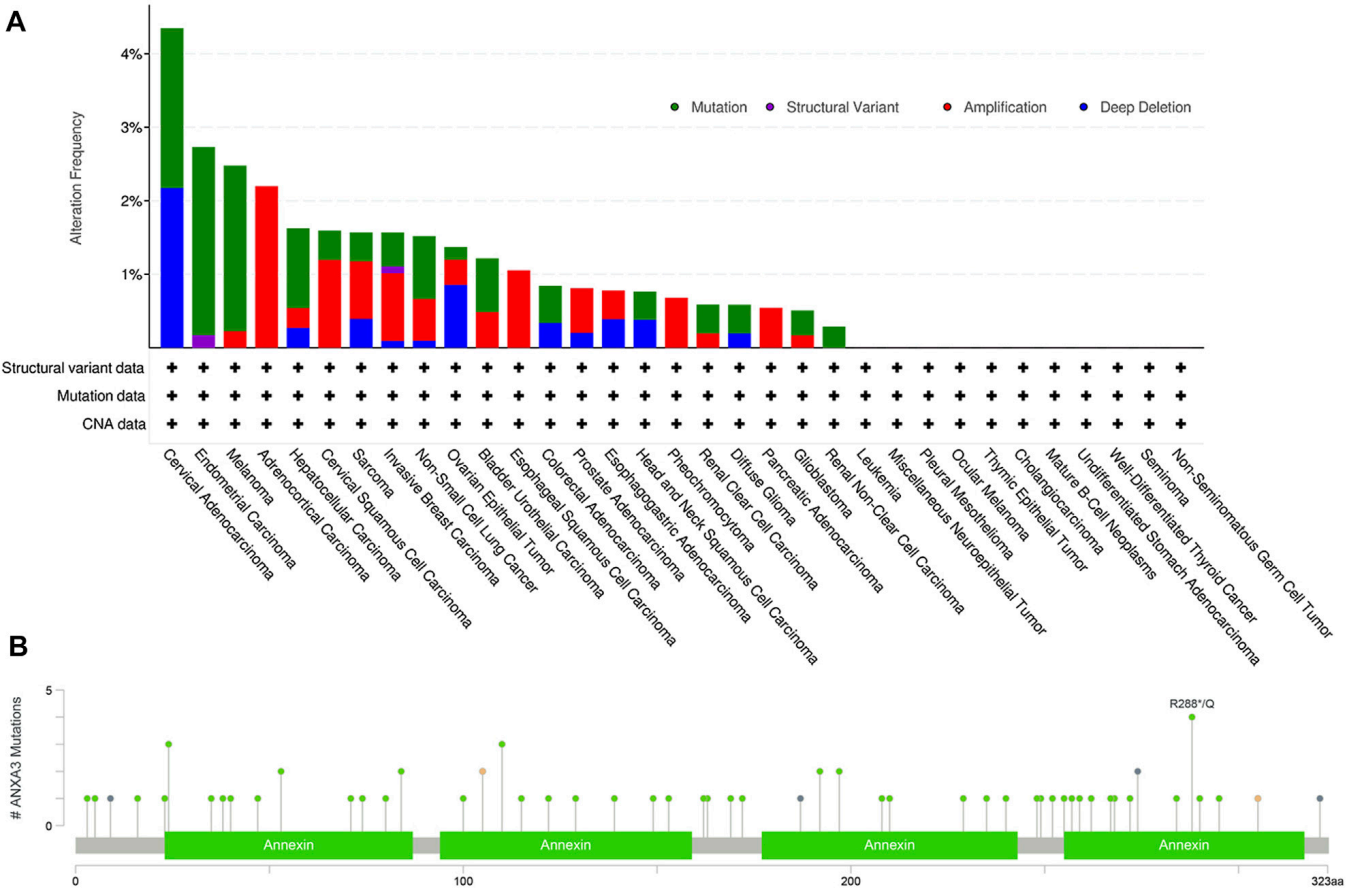
Genetic alterations of the ANXA3 gene. **(A)** The alteration frequency of the ANXA3 gene in different cancer types. Y-axis represented the alteration frequency, and X-axis showed the cancer types with descending alteration frequencies. The colors used in the histogram represented different types of genetic alterations, which were shown with the top right legend. **(B)** The type, location and number of mutations occurred on the ANXA3 gene. Y-axis showed the number of ANXA3 mutations and the X-axis represented the amino acid sequence. Green, black, orange, and violet dots represented missense mutations, truncating mutations, splice-site mutations, and gene fusions respectively. Data has been retrieved from cBioPortal (http://www.cbioportal.org, June 2021).

To generate a more comprehensive picture of the potentially oncogenic alterations on the ANXA3 gene, a computational analysis was performed using cBioPortal (http://www.cbioportal.org) querying 10,953 patients/10967 samples from 32 TCGA PanCancer Atlas studies (June 2021). Overall, the gene exhibited a low alteration frequency, with a detectable alteration in 121 (1.1%) of the total queried patients. The top three malignant diseases (Figure 3A) with the highest ANXA3 gene alterations are cervical adenocarcinoma (4.35% alterations including 2.17% mutation and 2.17% deep deletion), endometrial carcinoma (2.73% alterations including 2.56% mutation and 0.17% structural variant), and melanoma (2.48% alterations including 2.25% mutation and 0.23% amplification). Furthermore, the largest number of mutations on the ANXA3 gene (4 registered cases) occurred at amino acid 288 on its fourth annexin repeat domain, which could be either a nonsense mutation or a missense mutation replacing the arginine into glutamine (R288*/Q) (Figure 3B). Cancer types in which these mutations were detected are glioblastoma multiforme, uterine endometrioid carcinoma, and breast invasive ductal carcinoma. However, none of the mutations was further annotated with documented clinical implications from the literature, indicating that our understanding of their oncogenic effects is currently still highly rudimentary.

## The Role of ANXA3 in Anticancer Drug Resistance

**FIGURE 4:**
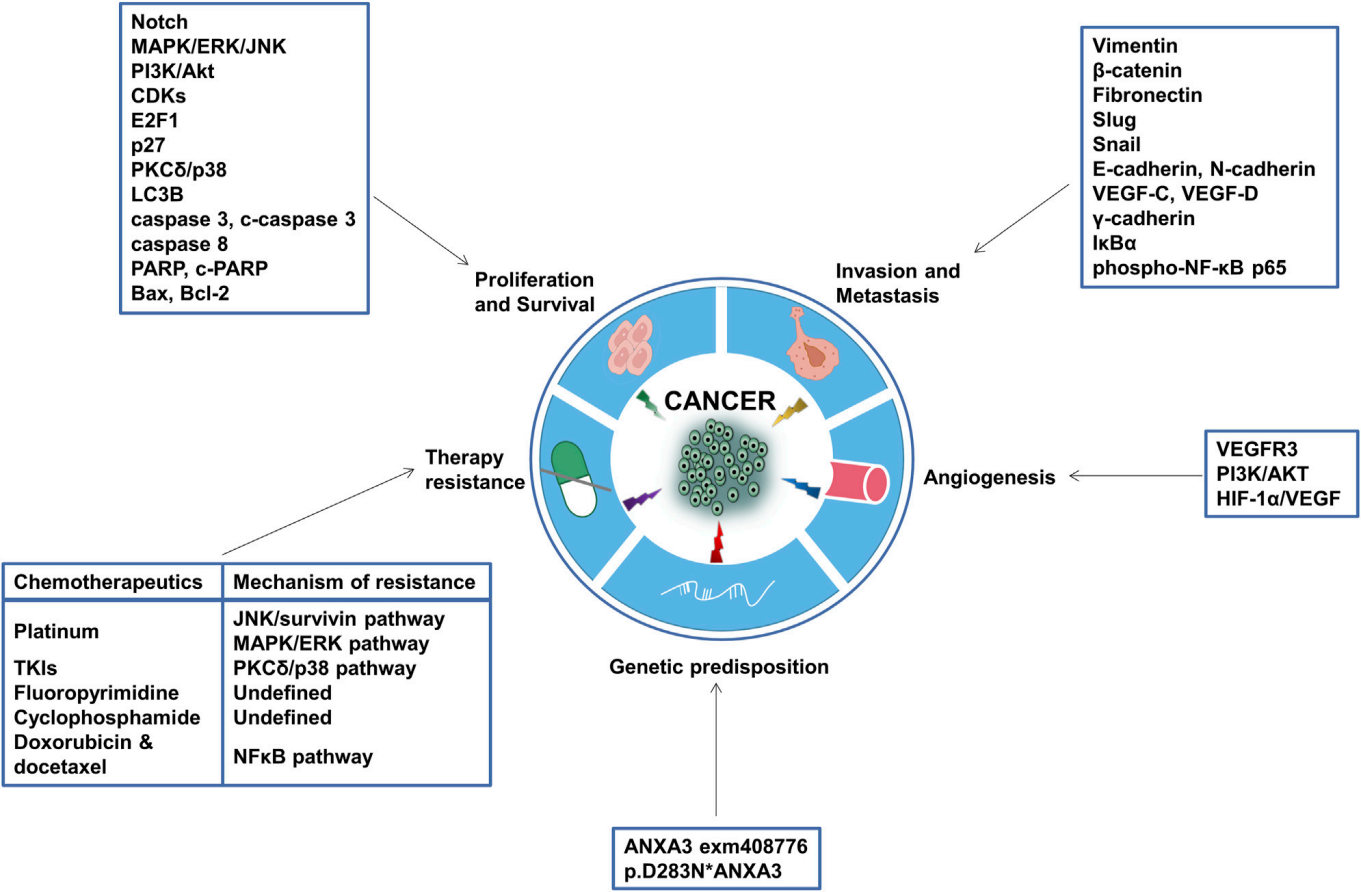
Tumorigenic functions of ANXA3. Aberrant expression of ANXA3 can exert multifaceted tumorigenic functions, such as promoting tumor cell proliferation, invasion, metastasis and angiogenesis, enhancing the propensity to form certain inherited tumors, and inducing resistance to chemotherapeutic agents. The putative mechanisms, target genes, effector proteins and pathways through which ANXA3 modulates these processes are annotated in the square text boxes. CDKs, Cyclin-dependent kinases; p27, Cyclin-dependent kinase inhibitor 1B; p38, p38 mitogen-activated protein kinases; LC3B, Microtubule-associated proteins 1A/1B light chain 3B; PARP, Poly (ADP-ribose) polymerase; bax, Bcl-2-associated X protein; bcl-2, B-cell lymphoma 2; VEGF, Vascular endothelial growth factor; IκBα, nuclear factor of kappa light polypeptide gene enhancer in B-cells inhibitor, alpha; NF-κB, nuclear factor kappa-light-chain-enhancer of activated B cells; VEGFR, Vascular endothelial growth factor receptor; HIF-1α, Hypoxia-inducible factor 1-alpha.

Chemotherapy is the standard-of-care treatment for a majority types of tumors, yet its efficacy is frequently plagued by the evolution of fatal drug resistance or temporary, fluctuating, or partial drug responses in patients. Based on the previously described tumorigenic characteristics of ANXA3, it is well-reasoned to hypothesize that ANXA3 also play a role in attenuating the vulnerability of cancer cells against chemotherapeutic drugs. As a result, in the past decade, investigators in this field have generated a substantial body of evidence delineating the involvement of ANXA3 in the development of anticancer drug resistance (Table 2 and Figure 4).

**TABLE 2:** The role of ANXA3 in therapy resistance.

Therapy resistance	Years	Tumor types	Main findings	References
Platinum resistance	2020	Non-small cell lung carcinoma	Anxa3-silencing siRNAs can eliminate oxaliplatin resistance in A549 NSCLC cells	Jin et al. (2020)
	2019	Non-small cell lung carcinoma	ANXA3 secreting CAF induces cisplatin resistance in NSCLC cells A549, H661 and SK-MES-1. The putative mechanism is the activation of JNK/survivin pathway	Wang et al. (2019)
	2019	Colorectal carcinoma	Depletion of ANXA3 in oxaliplatin resistant HCT116 and SW480 colorectal cancer cells inhibits the cell viability and BrdU incorporation, increases apoptosis and diminishes migration and invasion	Xu et al. (2019)
	2015	Hepatocellular carcinoma	Increasing resistance to cisplatin in ANXA3-overexpressing tumor cells *in vitro* and in mouse xenografts *in vivo*	Pan et al. (2015b)
	2012	Ovarian carcinoma	Lower intracellular accumulation and DNA binding of cisplatin and carboplatin in ANXA3 overexpressing ovarian cancer cells, accompanied by decreased p53 levels	Yin et al. (2012)
TKIs resistance	2018	Hepatocellular carcinoma cells	ANXA3 overexpression inhibits PKC/p38-mediated apoptosis and actives p38-mediated autophagy in sorafenib-resistant HepG2 and Huh7 HCC cells as well as in patient-derived xenografts	Tong et al. (2018)
	2013	45 cell lines	ANXA3 is associated with resistance against gefitinib, sorafenib, sunitinib and lapatinib	Pénzváltó et al. (2013)
Fluoropyrimidine resistance	2015	Hepatocellular carcinoma cells	Overexpressed ANXA3 significantly enhanced the IC50 of 5-FU in both cell-line models and mouse xenografts models	Pan et al. (2015b)
	2015	Gastric cancer	SNP rs2867461 in the ANXA3 gene is significantly correlated with the sensitivity against fluoropyrimidine	Takahashi et al. (2015)
Cyclophosphamide resistance	2013	Prostate cancer	ANXA3 expression in cyclophosphamide (CPA)-resistant PC3-D3 and PC3-D4 prostate cancer cells is higher compared to the chemo-sensitive wild type PC3 cell line	Kubisch et al. (2013)
	2010			Thoenes et al. (2010)
Doxorubicin and docetaxel	2018	Triple negative breast cancer	ANXA3 knockdown promotes the uptake of doxorubicin and sensitizes response to doxorubicin and docetaxel	Du et al. (2018)

***Platinum resistance.*** ANXA3 has been previously described as a potential marker for platinum sensitivity in patients with hepatocellular carcinoma (Pan et al., 2015b), ovarian carcinoma (Yan et al., 2010; Yin et al., 2012), non-small cell lung carcinoma (Wang et al., 2019; Jin et al., 2020) and colorectal carcinoma (Xu et al., 2019). In an attempt to ascertain the mechanism of platinum resistance in patients with hepatocellular carcinoma, researchers observed an enhanced degree of resistance to cisplatin-induced cell death in ANXA3-overexpressing tumor cells *in vitro* and in engrafted mice *in vivo* (Pan et al., 2015b). Support on this finding was provided by Yan et al., in which a significantly lower intracellular accumulation and DNA binding of cisplatin and carboplatin was detected in ANXA3 overexpressing ovarian cancer cells, accompanied by a decrement of the intracellular p53 level (Yan et al., 2010). Likewise, a substantially increased ANXA3 expression has been found in the serum of platinum-resistant ovarian cancer patients compared to the platinum sensitive group, which underscored the value of ANXA3 as a noninvasive marker for drug response prediction (Yin et al., 2012). Mechanistically, high level of ANXA3 released by CAFs has been confirmed to activate the JNK/survivin pathway in both cell-line models (A549, H661 and SK-MES-1) and mouse xenograft models of NSCLC, resulting in a markedly attenuated IC50 of cisplatin *in vitro* and an augmented xenograft tumor growth under cisplatin exposure *in vivo* (Wang et al., 2019). Conversely, abrogation of oxaliplatin resistance was achieved with ANXA3-silencing siRNAs in A549 NSCLC cells (Jin et al., 2020). Similarly, *in vitro* observations in oxaliplatin-resistant HCT116 and SW480 colorectal cancer cells further evidenced that ANXA3-knockdown has significantly impeded the phosphorylation of ERK and JNK, which led to decreased cell viability and BrdU incorporation as well as increased apoptosis and impaired migration and invasion abilities (Xu et al., 2019).

***TKIs resistance.*** In a microarray analysis testing the sensitivity of 45 different cancer cell lines to anticancer tyrosine kinase inhibitors (TKIs) targeting the ERBB/RAS pathway, ANXA3 was indicated to be associated with resistance against multiple TKIs including gefitinib, sorafenib, sunitinib, and lapatinib (Pénzváltó et al., 2013). Among these TKIs, only sorafenib resistance possesses a comprehensive *in vitro* and clinical evaluation, which is provided by the work of Tong et al. In this study, ANXA3 overexpression was detected in sorafenib-resistant HepG2 and Huh7 hepatocellular carcinoma cells as well as in patient-derived xenografts; conversely, inhibition of ANXA3 achieved with shANXA3 re-sensitized the response to sorafenib *in vitro* and limited tumor growth *in vivo* (Tong et al., 2018). The mechanistic rationale underlying this phenomenon was the inhibition of PKCδ/p38-mediated apoptosis and activation of p38-mediated autophagy, both of which are driven by a substantially elevated ANXA3 expression (Tong et al., 2018). Clinically, HCC patients with high levels of ANXA3 exhibited inferior outcomes under sorafenib treatment (Tong et al., 2018). Interestingly, investigators from this study further demonstrated that the combination of sorafenib with anti-ANXA3 monoclonal antibody effectively overcomes sorafenib resistance in both *ex vivo* organotypic cultures and patient-derived mouse xenografts (Tong et al., 2018), suggesting that targeting ANXA3 might be an actionable therapeutic approach for sorafenib-refractory HCC patients.

***Fluoropyrimidine resistance.*** The involvement of ANXA3 in 5-fluorouracil (5-FU) resistance has been previously reported in hepatocellular carcinoma, in which enforced ANXA3 expression established by ANXA3-overexpressing lentiviruses significantly enhanced the IC50 of 5-FU in both cell-line models and mouse xenografts models (Pan et al., 2015b). Furthermore, a bioinformatics analysis on the genomic data of 119 fluoropyrimidine-treated gastric cancer patients reported that SNP rs2867461 in the ANXA3 gene exhibited a significant correlation with the sensitivity to fluoropyrimidine treatment and might therefore serve as a potential genetic biomarker in predicting the therapeutic response (Takahashi et al., 2015).

***Cyclophosphamide resistance.*** Employing 2D-DIGE analysis, Thoenes et al. observed a significantly upregulated ANXA3 expression in cyclophosphamide (CPA)-resistant PC3-D3 and PC3-D4 prostate cancer cells compared to the chemo-sensitive wild type PC3 cell line (Thoenes et al., 2010). Moreover, western blot analysis validated the CPA-induced upregulation of ANXA3 in the isolated tumor tissues from mice that were engrafted with PC3-wt cells and subsequently exposed to a 70-day of metronomic CPA treatment (Thoenes et al., 2010). This finding is further consistent with the data from another microarray-based study on CPA-resistant PC3 tumors (Kubisch et al., 2013).

***Doxorubicin and docetaxel.*** The knockdown of ANXA3 has been demonstrated to promote the uptake of doxorubicin in human MDA-MB-231 breast cancer cells and 4T1 mouse mammary cancer cells (Du et al., 2018). MTT assay data from this study further indicated a substantially increased sensitivity of ANXA3-knockdown breast cancer cells to doxorubicin and docetaxel (Du et al., 2018). The reduction of the NF-κB signaling activity, which was shown to be a result of ANXA3-knockdown, was thought to be a putative mechanism underlying this improved drug response (Du et al., 2018). Despite these findings, current data is extremely limited regarding the role of ANXA3 in drug resistance against antineoplastic antibiotics and taxanes, which indicates the pressing need for further explorations in this field.

## The Diagnostic and Prognostic Value of ANXA3 in Cancer Treatment and Management

ANXA3 is one of the candidate biomarkers that has been extensively studied in different cancer models over the last decade. Collectively, studies have demonstrated that ANXA3 possesses potential diagnostic and prognostic value in the clinical treatment and management of an array of malignancies, including breast cancer (Pendharkar et al., 2016; Zhou et al., 2017b; Kim et al., 2018; Zhou et al., 2018), prostate cancer (Wozny et al., 2007; Köllermann et al., 2008; Schlomm et al., 2010; Guo et al., 2020), colorectal cancer (Marshall et al., 2010; Yip et al., 2010; Chang et al., 2014; Yang Q. et al., 2018) and some relatively rarer cancers such as bladder cancer (Hofmann et al., 2000), pancreatic cancer (Baine et al., 2011b) and testis cancer (Hofmann et al., 2000). In this section, we will summarize data from various studies which evaluated the clinical value of ANXA3 or provided novel clues on its diagnostic and prognostic potential.

***Breast cancer.*** Currently, stratifying patients into their correct subtypes and discriminating between early and late stages of these subtypes remain as a prime challenge in breast cancer (Pendharkar et al., 2016). This is of considerable importance for an optimal treatment design and patient outcome, especially in those patients with aggressive phenotypes such as triple-negative and basal-like breast cancer. Moreover, multiple shortcomings of the current mammography screening method have been reported, with the main concern remains on its suboptimal sensitivity in young women with dense breasts (Arif et al., 2015). Interestingly, the expression level of ANXA3 has been reported to be different per breast cancer subtype (Pendharkar et al., 2016; Zhou et al., 2017b; Kim et al., 2018; Zhou et al., 2018). Specifically, ANXA3 expression in basal subtype of breast cancer (MDA-MB-231, HCC-70, HCC-1954) was found to be significantly higher than other subtypes (Kim et al., 2018). Other studies further confirmed that a significantly higher expression of ANXA3 was detect in TNBC cells in comparison to luminal A and B subtypes (Zhou et al., 2017b; Zhou et al., 2018). Furthermore, employing 2D-DIGE and iTRAQ approaches, Pendharkar et al. demonstrated that ANXA3 upregulation is a marker for differentiating invasive ductal carcinoma with luminal B HER2 positive (LB) and HER2 enriched (HE) subtypes as well as their early and late stages (Pendharkar et al., 2016). These findings collectively confirmed the potential of ANXA3 in improving the accuracy of subtypes differentiation and risk stratification of breast cancer.

***Prostate cancer.*** The low explanatory power of existing clinical and histological parameters has also been reported in prostate cancer, where prognosis prediction relies solely on clinical stages, Gleason score and serum PSA and no molecular marker has been successfully translated into the routine clinical applications to date (Köllermann et al., 2008; Schlomm et al., 2010). The inferior performance of serum PSA in screening small neoplasms of initial stages (Köllermann et al., 2008) and the patient inconvenience from periodic biopsy sampling for Gleason score and pathological stage evaluation (Guo et al., 2020) further emphasize the pronounced clinical value of novel molecular biomarkers in prostate cancer. ANXA3 has been demonstrated to be downregulated in prostate cancer tissues (Wozny et al., 2007; Köllermann et al., 2008; Peraldo-Neia et al., 2011) but upregulated in the chemo-resistant PC3-D3 and PC3-D4 cell lines (Thoenes et al., 2010; Kubisch et al., 2013). Immunohistochemistry-based studies have reported a substantially less abundant ANXA3 staining in prostate cancer tissue when comparing with the surrounding epithelium and prostatic intraepithelial neoplasia (Wozny et al., 2007; Köllermann et al., 2008). In addition, the proportion of the ANXA3-negative tissue has been demonstrated to be correlated with advanced pathological stage and Gleason score as well as a reduced PSA-free survival (Köllermann et al., 2008). Using Kaplan-Meier analysis and multivariate cox regression, the study further indicated a significant association between ANXA3 staining abundance and biochemical relapse of prostate cancer (Köllermann et al., 2008). Support on this finding was provided by the work of Schlomm et al., in which ANXA3 was identified as an independent prognostic marker for the postoperative PSA recurrence (Schlomm et al., 2010). Of note, Schlomm et al. further demonstrated that incorporation of ANXA3 into the current risk stratification nomogram predicting the biochemical relapse after radical prostatectomy provided an enhancement of its predictive performance (AUC from 0.71 to 0.73) (Schlomm et al., 2010). Aside from postoperative recurrence risk, the value of ANXA3 in discriminating prostate cancer from benign tumors has also been elucidated by the work Guo et al., in which a 14-gene panel involves ANXA3 was constructed and evaluated on pre-biopsy urine and tissue specimens, displaying desirable predictive performance (AUC = 0.854) (Guo et al., 2020). Further evaluation of this gene predictor set on prospective and retrospective cohorts confirmed its ability in distinguishing low-risk patients from high-risk patients (Guo et al., 2020).

Unlike a wide spectrum of cancers overexpressing ANXA3, it is notable that the protein is downregulated in prostate cancer tissues. The putative mechanism underlying this downregulated expression pattern has been proposed in the context of autoimmune reactions in prostate cancer (Köllermann et al., 2008). Autoimmune antibodies against ANXA3 have been previously detected in the serum of prostate cancer patients (Köllermann et al., 2008), which was thought to be a result of the release of ANXA3 by prostatic epithelium cells in the form of prostasomes (Wozny et al., 2007; Köllermann et al., 2008). These granules, which are secreted by both normal and malignant prostate epithelium, have been broadly considered to harbor a strong immunogenicity (Feist et al., 2000; Minelli et al., 2005; Larsson et al., 2006). Studies have shown that, compared to patients with benign prostate hyperplasia and other noncancerous prostate disorders, prostate cancer patients carried significantly higher levels of anti-prostasome antibodies in their blood (Feist et al., 2000; Minelli et al., 2005; Larsson et al., 2006). This finding has linked anti-prostasome antibody titer to malignant transformation. It was therefore postulated that some prostate tumors could induce the formation of autoimmune antibodies against ANXA3 to neutralize its tumor-suppressive effects (Köllermann et al., 2008). This speculation also explained why decreased ANXA3 expression is associated with unfavorable clinicopathological features in prostate cancer patients (Köllermann et al., 2008). Nevertheless, the functional rationale of the antitumor activity of ANXA3 in prostate cancer, which is inconsistent to its tumorigenic roles in other malignancies, still necessitates additional experimental analyses.

***Colorectal cancer.*** Colorectal cancer (CRC) is another well-known malignancy that can benefit most from early detection via population screening, since almost every colorectal tumor arises from a benign adenomatous polyp and is easily surgically resectable once detected (Marshall et al., 2010; Yang Q. et al., 2018). However, the unpleasant and inconvenient nature of current CRC screening procedures has led to a low compliance in screening participation (Kronborg, 2004; Marshall et al., 2010), which results in a higher incidence of advanced tumors and metastases. In an attempt searching for novel tumor-specific immunogens in CRC, mass spectrometry analyses have reproducibly detected the presence of ANXA3 in the tumor protein extracts blotted with patient’s own sera (Yang Q. et al., 2018). Further verifications using immunohistochemistry and western blot assays confirmed the increased ANXA3 expression in tumor tissues compared to non-tumor tissues (Yang Q. et al., 2018). These findings collectively indicated the potential of ANXA3 as a novel serum antibody screening marker in CRC. Additionally, a series of microarray-based studies have designed novel gene predictor sets that can potentially contribute to improving the performance of the existing screening methods and overcoming the current low screening compliance (Marshall et al., 2010; Yip et al., 2010; Chang et al., 2014). A blood-based 7-gene panel (ANXA3, CLEC4D, LMNB1, PRRG4, TNFAIP6, VNN1, and IL2RB) developed by Marshall et al. exhibited good predictive performance in discriminating CRC from controls (AUC = 0.80) and in stratifying patients’ current relative risk (Marshall et al., 2010). This gene predictor set was further validated in a Malaysian cohort, showing consistent results (Yip et al., 2010). In a study published in 2014, Chang et al. evaluated 17 previously described CRC-associated genes (including those from Marshall et al.) and further constructed a novel blood-based 7-gene model comprised of CpEB4, EIF2S3, MGC20553, MS4A1, ANXA3, TNFAIp6 and IL2RB, which showed a more superior performance in logistic regression analyses (AUC = 0.99) (Chang et al., 2014).

***Other cancers.*** With the hypothesis that peripheral blood mononuclear cells (PBMCs) act as the first line of defense against early emerged neoplastic cells and thereby capable of more accurately representing the tumor biology in initial stages, Baine et al. investigated the differentially expressed genes in PBMCs of patients with pancreatic cancer (PC) and found that ANXA3 was significantly upregulated in PC patients compared to healthy controls (Baine et al., 2011b). The study further established a blood-based 7-gene predictor panel (ANXA3, SSBP2, Ube2b-rs1, CA5B, F5, TBC1D8, ARG1, and ADAMTS20) with a sensitivity of 83% and specificity of 75% in discriminating PC patients (Baine et al., 2011b). In a LC−MRM/MS analysis of urine samples from 30 bladder cancer patients and 89 non-cancer patients, ANXA3 was most frequently detected in bladder cancer samples (Tsai et al., 2018). Validation using western blot analysis further confirmed the elevated level of ANXA3 in both urine and tumor specimens from bladder cancer patients (Tsai et al., 2018). In another bioinformatics study based on previously published microarray data, a significantly augmented expression of the ANXA3 gene was detected in testicular carcinoma *in situ* (CIS) samples and further validated using RT-qPCR, suggesting the potential of ANXA3 serving as a novel clinicopathological marker for testicular CIS (Almstrup et al., 2007).

## Conclusion

Cancer is a major public health issue and a highly lethal disease worldwide. The high mortality rate of many cancers can be attributed to the evasion of detection of benign or preneoplastic tumors, the occurrence of intrinsic or acquired therapy resistance, and the suboptimal predictive power of the routinely used screening techniques and clinicopathological parameters. Emerging evidence has suggested that ANXA3 might be a promising biomarker candidate and an attractive therapeutic target that harbors the potential to address these obstacles. According to the gathered data in this review, differential expression of ANXA3 has been demonstrated in a wide array of cancers, which is capable of sustaining cell proliferation signaling, promoting invasion and metastasis, inducing angiogenesis and resistance to various chemotherapeutic agents. Genetic alterations in the ANXA3 gene have also been reported to be associated with an enhanced tumor susceptibility. As a candidate molecular marker, ANXA3 exhibited pronounced clinical value in risk stratification, early detection, patient differentiation and active surveillance. Nonetheless, some limitations to the current findings and conclusions still need to be noted. Despite the substantial body of evidence on its tumorigenic role, the precise upstream and downstream signaling transduction of ANXA3 remains incompletely elucidated, and there is a lack of mechanistic studies on the role of ANXA3 in highly prevalent cancers. Besides, the underlying mechanisms of ANXA3-induced chemoresistance are not fully understood, and investigations so far have not covered all chemotherapeutic drugs, nor other anticancer therapies, such as radiotherapy. Furthermore, most of the studies evaluating the diagnostic and prognostic value of ANXA3 suffer from small sample sizes, which indicates that more conclusive data from larger patient cohorts are still urgently awaited. Therefore, the eventual clinical implementation of ANXA3 as a therapeutic target or as a diagnostic or prognostic biomarker still requires further investigations to 1) elucidate the complete picture of the upstream and downstream signaling pathways of ANXA3; 2) obtain deeper insights into the mechanisms underlying ANXA3-induced chemoresistance as well as the role of ANXA3 in other types of anticancer therapy resistance such as radioresistance; and 3) evaluate the clinical significance of ANXA3 as a diagnostic or prognostic biomarker on larger patient cohorts.

## Abbreviations

ANXA3, Annexin A3; Bax, Bcl-2-associated X protein; Bcl-2, B-cell lymphoma 2; CAF, Cancer associated fibroblast; CDKs, Cyclin-dependent kinases; CRC, Colorectal cancer; EMT, Epithelial-Mesenchymal Transition; HCC, Hepatocellular carcinoma; HIF-1α, Hypoxia-inducible factor 1-alpha; IκBα, Nuclear factor of kappa light polypeptide gene enhancer in B-cells inhibitor, alpha; LC3B, Microtubule-associated proteins 1A/1B light chain 3B; MET, Mesenchymal-epithelial transition; NF-κB, Nuclear factor kappa-light-chain-enhancer of activated B cells; NSCLC, Non-small cell lung cancer; p27, Cyclin-dependent kinase inhibitor 1B; p38, p38 mitogen-activated protein kinases; PARP, Poly (ADP-ribose) polymerase; SNP, Single-nucleotide polymorphism; TKI, Tyrosine kinase inhibitor; TNBC, Triple-negative breast cancer; TNF, Tumor necrosis factor; VEGF, Vascular endothelial growth factor; VEGFR, Vascular endothelial growth factor receptor.
